# Uma Iniciativa Nacional de Melhoria da Qualidade em Cardiologia: O Programa Boas Práticas em Cardiologia no Brasil

**DOI:** 10.36660/abc.20230703

**Published:** 2023-11-14

**Authors:** Henrique Tria Bianco

**Affiliations:** 1 Universidade Federal de São Paulo São Paulo SP Brasil Universidade Federal de São Paulo, São Paulo, SP – Brasil

**Keywords:** Cardiologia, Melhoria de Qualidade, Prática Clínica Baseada em Evidências

O sistema de saúde público no Brasil se ocupa da alta demanda de atendimento, atuando na prevenção primária, secundária e terciária. A despeito de inúmeros esforços e iniciativas e considerando a melhoria destes atendimentos, os resultados ainda estão aquém das necessidades, carecendo de implementação de novas frentes de ação.^1^Adicionalmente, pouco tem sido executado para o melhor controle e conhecimento da utilização dos recursos destinados à saúde, impactando em barreiras para a adoção de terapias baseadas em evidências. A mortalidade hospitalar por doenças cardiovasculares no Brasil é ainda alta, e programas robustos de melhoria da qualidade são desejáveis e necessários.

Dois ensaios randomizados realizados no Brasil (BRIDGE-ACS e IMPACT-AF) para testar intervenções multifacetadas para melhorar a adesão às recomendações de diretrizes mostraram que a implementação de intervenções de melhoria da qualidade (MQ) é viável e pode ser eficaz.^2 , 3^

Dentro deste cenário, destacamos o Programa Boas Práticas em Cardiologia (BPC no Brasil), que visa o incremento de ações no cuidado na síndrome coronária aguda (SCA), na insuficiência cardíaca (IC), e na fibrilação atrial (FA).^4^ Este importante programa descreveu com primor as características populacionais, o tratamento hospitalar e os desfechos de pacientes admitidos em hospitais públicos no Brasil, avaliando a efetividade dos programas de qualidade assistencial, baseados em diretrizes e recomendações. Desta forma, com importante contribuição, neste artigo podemos observar os resultados deste projeto, vendo a melhoria nas taxas destes desfechos cardiovasculares no Brasil. Um total de 12.167 pacientes com diagnóstico de SCA, IC, ou FA foram incluídos, provenientes de 19 instituições de diferentes regiões brasileiras.

As medidas de desempenho foram delineadas para avaliar a qualidade do tratamento de pacientes com SCA, IC e FA. As medidas foram desenvolvidas de acordo com as diretrizes da Sociedade Brasileira de Cardiologia e do *American College of Cardiology /American Heart Association* . Medidas de desempenho para cada condição crítica foram analisadas para cada centro antes e após a participação no programa BPC. Como resultados, observou-se uma taxa de prescrição de AAS (ácido acetil salicílico) de 96,2%, que é comparável a outros países, como o Reino Unido (98,1%) e a Suécia (94,6%). Ainda, a taxa de uso de betabloqueadores por ocasião da alta hospitalar foi 88,6%, também comparável às taxas dos países supracitados.^5 - 7^

Um aspecto de fundamental importância refere-se aos resultados obtidos na qualidade de vida dos pacientes com IC, uma vez que esse é um preditor de desfechos clínicos adversos, tais como mortalidade em curto prazo e reinternação precoce desses pacientes. No presente estudo, foi possível observar que intervenções farmacológicas e não farmacológicas melhoram a qualidade de vida em pacientes com IC após seis meses da alta hospitalar.

Um interessante estudo de intervenção educativa multifacetada e multinível, destinada a melhorar o uso de anticoagulação oral (ACO) em pacientes com FA e em risco de acidente vascular cerebral (AVC), resultou em aumento significativo na proporção de pacientes tratados, com potencial de melhorar a prevenção do AVC.^8^Entretanto, apesar da existência de tratamentos farmacológicos seguros e eficazes para a prevenção do AVC entre pacientes com FA, apenas 40% a 60% dos pacientes estavam sob tratamento regular. Ainda, apenas dois terços dos pacientes com FA que tiveram um AVC estavam tomando ACO no momento do evento agudo.^9 - 11^Assim, existe uma necessidade médica não atendida de estudos que desenvolvam intervenções baseadas em evidências, e que possam levar a um maior uso de ACO em pacientes com FA que correm risco de AVC.^12^

O ACCEPT, um estudo observacional prospectivo incluiu pacientes internados com diagnóstico de SCA em 47 hospitais brasileiros. Os pacientes foram seguidos por 1 ano e coletaram-se dados sobre prescrição médica e ocorrência de eventos cardiovasculares maiores. A prescrição completa de terapias baseadas em evidência na admissão hospitalar foi de apenas 62%, mostrando a necessidade de elaboração de estratégias para melhorar o uso de terapias específicas, no sentido de minimizar os eventos cardiovasculares na população brasileira.^13^

## Conclusões

O programa BPC determina e mensura as métricas de qualidade assistencial, pautado por diretrizes especializadas, aplicáveis no manejo de algumas doenças cardiovasculares, em destaque para a FA, a IC e SCA.
