# Supplementary material for: Uma Iniciativa Nacional de Melhoria da Qualidade em Cardiologia: O Programa Boas Práticas em Cardiologia no Brasil
Source: Arq Bras Cardiol. 2023 Nov 14;120(10):e20230703. [Article in Portuguese] doi: 10.36660/abc.20230703 (PMC10697683; doi:10.36660/abc.20230703)
Supplement: Translation

# Translation: A National Quality Improvement Initiative in Cardiology: The Good Practices in Cardiology Program in Brazil

**Authors:** Henrique Tria Bianco

**Affiliations:** 1 Brazil Universidade Federal de São Paulo, São Paulo, SP – Brazil

**Keywords:** Cardiology, Quality Improvement, Evidence-Based Practice

The public health system in Brazil deals with the high demand for care, acting in primary, secondary, and tertiary prevention. Despite countless efforts and initiatives and considering the improvement of these services, the results are still below the needs, requiring the implementation of new fronts of action.^1^Additionally, little has been carried out to better control and understand the use of resources destined for health, impacting barriers to adopting evidence-based therapies. Hospital mortality from cardiovascular diseases in Brazil is still high, and robust quality improvement programs are desirable and necessary.

Two randomized trials conducted in Brazil (BRIDGE-ACS and IMPACT-AF) to test multifaceted interventions to improve adherence to guideline recommendations showed that implementing quality improvement (QI) interventions is feasible and can be effective.^2 , 3^

Within this scenario, we highlight the Good Practices in Cardiology Program (BPC in Brazil), which aims to increase actions in care for acute coronary syndrome (ACS), heart failure (HF), and atrial fibrillation (AF).^4^This important program exquisitely described the population characteristics, hospital treatment, and outcomes of patients admitted to public hospitals in Brazil, evaluating the effectiveness of quality care programs based on guidelines and recommendations. Thus, with an important contribution in this article, we can observe the results of this project, seeing the improvement in the rates of these cardiovascular outcomes in Brazil. Twelve thousand one hundred sixty-seven patients diagnosed with ACS, HF, or AF were included from 19 institutions in different Brazilian regions.

Performance measures were designed to assess the quality of treatment for patients with ACS, HF, and AF. The measures were developed following the Sociedade Brasileira de Cardiologia and the American College of Cardiology/American Heart Association guidelines. Performance measures for each critical condition were analyzed for each center before and after participation in the BPC program. As a result, a prescription rate for ASA (acetylsalicylic acid) of 96.2% was observed, which is comparable to other countries, such as the United Kingdom (98.1%) and Sweden (94.6%). Furthermore, the rate of beta-blocker use at the time of hospital discharge was 88.6%, also comparable to the rates in the countries mentioned above.^5 - 7^

An aspect of fundamental importance refers to the results obtained in the quality of life of patients with HF since this is a predictor of adverse clinical outcomes, such as short-term mortality and early readmission of these patients. In the present study, it was possible to observe that pharmacological and non-pharmacological interventions improve the quality of life in patients with HF six months after hospital discharge.

An interesting multifaceted, multilevel educational intervention study aimed at improving the use of oral anticoagulation (OAC) in patients with AF and at risk of stroke resulted in a significant increase in the proportion of patients treated, with the potential to improve stroke prevention.^8^However, despite the existence of safe and effective pharmacological treatments for stroke prevention among patients with AF, only 40% to 60% of patients were under regular treatment. Still, only two-thirds of patients with AF who had a stroke took OCs at the time of the acute event.^9 - 11^Thus, there is an unmet medical need for studies that develop evidence-based interventions that could lead to greater use of OAC in patients with AF who are at risk of stroke.^12^

ACCEPT, a prospective observational study, included patients admitted with a diagnosis of ACS in 47 Brazilian hospitals. Patients were followed for 1 year, and data on medical prescriptions and the occurrence of major cardiovascular events were collected. The complete prescription of evidence-based therapies upon hospital admission was only 62%, showing the need to develop strategies to improve the use of specific therapies to minimize cardiovascular events in the Brazilian population.^13^

## Conclusions

The BPC program determines and measures care quality metrics, guided by specialized guidelines, applicable in managing some cardiovascular diseases, especially AF, HF, and ACS.
